# Natural resistance to Potato virus Y in *Solanum tuberosum* Group Phureja

**DOI:** 10.1007/s00122-019-03521-y

**Published:** 2020-01-16

**Authors:** Lesley Torrance, Graham H. Cowan, Karen McLean, Stuart MacFarlane, Aqeel N. Al-Abedy, Miles Armstrong, Tze-Yin Lim, Ingo Hein, Glenn J. Bryan

**Affiliations:** 1 The James Hutton Institute, Invergowrie, Dundee, DD2 5DA UK; 2 The School of Biology, The University of St Andrews, St Andrews, KY16 9ST UK; 3 Plant Protection Department, College of Agriculture, University of Kerbala, Kerbala, Iraq; 4 Columbia University, New York, NY 10027 USA; 5 Division of Plant Sciences at the James Hutton Institute, School of Life Sciences, University of Dundee, Dundee, DD2 5DA UK

## Abstract

**Key Message:**

Novel major gene resistance against *Potato virus Y* in diploid populations of *Solanum tuberosum* Groups Phureja and Tuberosum was biologically and genetically characterised. Named Ry(o)_phu_, it mapped to chromosome 9.

**Abstract:**

A new source of genetic resistance derived from *Solanum tuberosum* Group *Phureja* against *Potato virus Y* (PVY) was identified and genetically characterised in three diploid biparental potato populations. Segregation data for two populations (05H1 and 08H1) suggested the presence of a single dominant gene for resistance to PVY which, following DaRT analysis of the 08H1 cross, was mapped to chromosome 9. More detailed genetic analysis of resistance utilised a well-characterised SNP-linkage map for the 06H1 population, together with newly generated marker data. In these plants, which have both *S. tuberosum* Group *Phureja* and *S. tuberosum* Group *Tuberosum* in their pedigree, the resistance was shown to map to chromosome 9 at a locus not previously associated with PVY resistance, although there is evidence for at least one other genetic factor controlling PVY infection. The resistance factor location on chromosome 9 (named as Ry(o)*phu*) suggests a potential role of NB-LRR genes in this resistance. Phenotypic analysis using a GUS-tagged virus revealed that a small amount of PVY replication occurred in occasional groups of epidermal cells in inoculated leaves of resistant plants, without inducing any visible hypersensitive response. However, the virus did not enter the vascular system and systemic spread was completely prevented.

**Electronic supplementary material:**

The online version of this article (10.1007/s00122-019-03521-y) contains supplementary material, which is available to authorized users.

## Introduction

*Potato virus Y* (PVY), the type species of the Genus *Potyvirus*, is one of the most important viral pathogens of potato worldwide and is economically damaging in other Solanaceous crops such as pepper, tomato and tobacco (Scholthof et al. 2011). Recombinant viruses derived from the ordinary (PVY^O^) and necrotic (PVY^N^) strains are increasing in incidence and in some cultivars these strains cause tuber damage making them unmarketable (Gray et al. 2010; Karasev et al. 2011; Kamangar et al. 2014). Moreover, recombinant PVY strains have been shown to overcome mature plant resistance (MPR; Kumar, Roberts and Torrance, personal communication), a form of resistance often relied on, for example, in Northern Europe, where virus vector aphids arrive later in the growing season after MPR has developed in the crop (Beemster 1987). PVY is the predominant virus problem in most potato production systems worldwide, and effective control methods are urgently required.

In most regions with well-developed potato production systems, the health of seed potatoes used for producing the ‘ware’ or table crop is strictly regulated. Seed tubers are produced from virus-free stocks, and the health of seed stocks is closely monitored during field multiplications, as a result breeding for virus resistance in some countries has been of lower priority than for other biotic traits such as resistance to late blight or nematodes. However, environmental change is resulting in milder winters, allowing increased survival of vector aphids and earlier build up and migration of populations. Moreover, insecticide treatment is not effective in controlling PVY and other viruses that are spread in the non-persistent manner by aphids, because aphids can transmit the virus before the insecticides take effect. These circumstances have increased the importance of deploying host resistance.

Host resistance is the most effective way to control PVY, and sources of dominant resistance have been reported in wild and cultivated species of potato. Examples of such host resistances are Ry_sto_ from *Solanum stoloniferum*, chromosome 12 (Flis et al. 2005; Song et al. 2005), Ry_adg_ from *S. tuberosum* Group andigena, chromosome 11 (Hämäläinen et al. 1997, 1998) and Ry_chc_ from *S. chacoense*, chromosome 9 (Sato et al. 2006). Recently, the Ry_sto_ gene was isolated from the dihaploid clone Alicja and found to encode a nucleotide-binding leucine-rich repeat (NB-LRR) protein with an N-terminal TIR domain, a structure common to many different plant pathogen resistance proteins (Grech-Baran et al. 2019). Expression of this protein in transgenic *S. tuberosum* cultivars (cvs Maris Piper and Russet Burbank) rendered these plants resistant to PVY infection, thus, demonstrating the usefulness of research to identify and map PVY resistance genes from different sources.

*Solanum tuberosum* Group Phureja (Phureja) potatoes were favoured by early Andean farmers for their lack of dormancy and fast tuber development, so that they could be used to produce crops up to three times per year in the Andean valleys (Bradshaw and Ramsay 2009). In the UK during the 1970s, a diploid mass-selection scheme was initiated that crossed edible diploid potatoes from the *S. tuberosum* groups Phureja and Stenotomum by open pollination in the field (Carroll 1982). Over time this material was selected for resistance to various diseases and other properties such as tuberisation under long days (Carroll 1982; De Maine et al. 1993; Bradshaw et al. 2006). From these selections, commercial cultivars such as Mayan Gold and Inca Dawn were released. We have previously tested nearly forty of these Phureja clones and found some of them to be resistant to various PVY strains (PVY^o^, PVY^C^, PVY^N^ and PVY^NTN^) as well as to PVV and PVA (Torrance et al. 2009). The diagnostic molecular markers published for Ry_sto_ and Ry_adg_ resistances to PVY (Kasai et al. 1999; Flis et al. 2005; Song et al. 2005) failed to show genetic linkage to resistance in Phureja and Stenotomum crosses suggesting that the observed resistances are genetically distinct to those previously described (Torrance et al. 2009). In this report, we present a detailed genotypic and biological analysis of this novel form of potyvirus resistance. In performing this analysis, we make use of both a dense SNP-based linkage map (Prashar et al. 2014) as well as RenSeq, a target enrichment, next generation sequencing (NGS)-based bulked segregant analysis that focusses on NB-LRR genes (Jupe et al. 2013).

## Materials and Methods

### Potato clones and populations

Potato clones were grown from tubers and multiplied by stem cuttings to give enough material for replicated virus challenges. Three populations, 05H1, 08H1 and 06H1, were used for mapping. The 05H1 F1 progeny were obtained from a cross between *S. tuberosum* Group *Phureja* parents DB257(28) and 84.2P.75. The 08H1 F1 progeny were obtained from a cross between *S. tuberosum* Group *Phureja* parents DB375(1) and 84.2P.75. The 06H1 progeny were obtained from a cross between parents HB171(13) (parentage PDH247 × DB226(70)) and 99.FT.1b5 (parentage 2DH40(3) × DB337(37)), both parents being derived from crosses between diploids of *Solanum tuberosum* Group *Tuberosum* and *Solanum tuberosum* Group *Phureja* (Prashar et al. 2014). Individual clones from potato populations were propagated by stem cuttings and maintained in an insect-proof glasshouse maintained at 22 °C day/14 °C night with supplementary lighting to achieve a 16 h day length.

### Phenotyping of potato clones

A Scottish isolate of PVY^o^ (Accession AJ585196.1) held in the virus collection at the James Hutton Institute was maintained in *Nicotiana tabacum* cv. White Burley. Other PVY isolates used in this study were from the Hutton collection (SCRI) or from Science and Advice for Scottish Agriculture (SASA): PYV^N^ [SCRI; Accession AJ585197.1; and SASA 149], PVY^NTN^ [SASA 390], PVY^N-Wi^ [SASA 207; Accession AJ5848561.1]. Inocula were prepared by macerating PVY-infected *N. tabacum* leaves in water and were manually inoculated onto the leaves of carborundum-dusted potato plants. Virus was detected in upper non-inoculated leaves using a double antibody sandwich enzyme-linked immunosorbent assay (ELISA) using PVY-all antibodies (SASA, Edinburgh, UK), following the method described by Torrance (1992). Briefly, leaf samples were collected 21 days post-inoculation and macerated with extraction buffer (1 g/10 ml; 0.07 M phosphate buffered saline containing polyvinyl pyrrolidone at 2% w/v and 0.05% v/v Tween 20). Absorbance values (A_405nm_) were recorded after incubation of substrate for 1 h at room temperature (22 °C) and after 16 h at 4 °C using an ELISA microplate reader (Multiskan® Ascent). Values that exceeded twice the mean control values of non-infected potato leaves were considered positive for virus infection (Fenlon and Sopp 1991).

### PVY GUS infections

Cultures of *Agrobacterium tumefaciens* strain LBA4404 transformed with the infectious PVY N605 clone expressing β-glucuronidase (PVY-GUS; kindly provided by E. Johansen) were used to infiltrate leaves of *Nicotiana benthamiana* plants. After 10–14 days, the infiltrated leaves were used as a source of inoculum for manual inoculation of carborundum-dusted potato leaves as described above. Three weeks post-inoculation, inoculated and non-inoculated apical leaves were collected and assayed for GUS activity as follows: Leaves were vacuum infiltrated in X-gluc (5-bromo-4-chloro-3-indolyl b-d-glucuronide) substrate solution (1 mg/ml X-gluc in 0.1 M NaH_2_PO_4_ and 0.1 M Na_2_HPO_4_ pH 7, 0.02% Triton X-100, 0.5 mM potassium ferricyanide, 0.5 mM potassium ferrocyanide) and incubated at 37 °C in the dark overnight (approx. 16 h). The leaves were rinsed in water and stored in 70% ethanol to decolourise and GUS expression, which is a marker of the extent and location of the PVY infection, was then recorded photographically.

### DNA isolation from plants

Leaf material was collected from glasshouse grown plants of the 06H1 and 08H1 populations and stored frozen. DNA was extracted from 100 mg of frozen leaf material using a Qiagen DNeasy plant mini kit (Qiagen) and quantified using a Nanodrop 1000 spectrophotometer (Thermo Scientific).

### PCR

Genomic sequences were amplified in 20 µl PCR reactions containing 20 ng of template DNA using Roche Taq polymerase and buffer following the manufacturer’s instructions. The standard PCR conditions used were 3 min at 94 °C, then 35 cycles of 15 s at 94 °C, 30 s at 55 °C and 30 s at 72 °C, followed by a final extension of 5 min at 72 °C. The 55 °C annealing temperature was modified, where necessary, for individual primer pairs in order to optimise the PCR product generated. PCR products were analysed by electrophoresis on agarose gels in TBE buffer and visualised by ethidium bromide staining. PCR primers were designed using PRIMER3 (Untergasser et al. 2012). Primer sequences are provided in Supplementary Table 1.

### Genetic Marker Development

The simple sequence repeat (SSR) markers deployed in this work have previously been described by Milbourne et al (1998). One primer from each pair was synthesised with the 5′ end-nucleotide labelled with 6-FAM to enable detection on an ABI3730 sequencer. SSR products were visualised using GeneMapper software (ABI) as in Macaulay et al. (2001). Rox500 (ABI) was used as an internal size standard to enable automatic sizing of the peaks. Additional SSR and sequence-tagged sites (STS) markers were selected from the SOL Genomics Network (https://solgenomics.net), and other candidate markers were designed based on the potato genome sequence superscaffold and pseudomolecule information (Sharma et al. 2013). PCR products were sequenced using an Applied Biosystems Big Dye Terminator cycle sequencing reaction kit (PE Biosystems, Warrington, UK), and the specific primers used to generate the original PCR product. Samples were run on an ABI3730 automated sequencer (PE Bio-systems, La Jolla, California, USA) and sequences edited and aligned using Sequencher software (Gene Codes Corporation).

### Mapping and QTL analysis

Markers were added to the existing 06H1 linkage map (Prashar et al. 2014) using JoinMap 4 (van Ooijen 2006). QTL mapping was performed using MapQTL® 6.0 (Van Ooijen 2009) and Genstat 18 (VSN International Ltd.). The nonparametric Kruskal–Wallis test supported in MapQTL version 6.0 was performed. In this method, a single marker analysis was used to test the association of a marker with the trait at significance *P* ≤ 0.001. The identified QTL regions were further explored by using single trait single environment QTL analysis using Genstat 19 (VSN International Limited). This was done using composite interval mapping (CIM) controlling the effects of chromosomes onto the QTL being tested and so increasing the precision of QTL detection (Zeng 1994).

### Renseq

RenSeq target enrichment and sequencing was performed according to Jupe et al. (2013) with minor modifications as detailed in Chen et al. (2018). Sequencing was conducted on an Illumina MiSeq platform using the v3 2 × 300 bp kit (Illumina). Quality control of reads, on-target rate calculations and SNP filtering were as described in Chen et al. (2018).

## Results

### Initial phenotypic characterisation of diploid potato populations

Previously, we challenged a collection of 39 *S. tuberosum* Group Phureja and Group Stenotomum clones by mechanical inoculation with PVY^O^ and found that 28 clones were completely resistant, with no symptoms induced and no virus was detected by ELISA in the upper, non-inoculated leaves. Only six clones displayed a hypersensitive response with necrotic lesions observed in the inoculated leaves but the plants did not become systemically infected, and five clones were susceptible to systemic infection (Torrance et al. 2009).

In this study, we have extended the previous survey of diploid Group Phureja germplasm by examining three distinct diploid populations. Two populations are pure Phureja populations: 05H1, derived from a cross between resistant clone DB257(28) and susceptible clone 84.2P.75, and 08H1, derived from a cross between resistant clone DB375(1) and susceptible clone 84.2P.75. Thereafter, a diploid Tuberosum-Phureja population, 06H1, derived from the cross between resistant clone HB171(13) and susceptible clone 99.FT.1b5 (Prashar et al. 2014) was also examined. These populations were established separately for different purposes but are also suitable for the analysis of PVY resistance in *S. tuberosum* Group Phureja. The populations were examined to obtain PVY susceptibility and genotypic data for detailed phenotypic and genetic analysis of the segregating resistance gene(s).

### Phenotypic analysis of 05H1, 08H1and 06H1 populations

In initial tests, the susceptible parent (84.2P.75) of the 05H1 population was found to be susceptible to infection by three PVY strains (PVY^o^, PVY^NTN^ and PVY^N-Wi^), whereas the resistant parent (DB257(28) of this cross was not infected. Thereafter, challenge inoculations and subsequent ELISA of upper, non-inoculated leaves of the progeny of this cross showed 59 resistant and 44 susceptible clones, corresponding to an approximate 1:1 segregation ratio (X^2^ = 2.18, *P* > 0.1), suggesting the presence of a single dominant resistance gene in the heterozygous configuration in the resistant parent DB257(28).

Prior to testing of the 08H1 population, the parental clones 842.P75 (susceptible) and DB375(1) (resistant) were assessed for their reaction to four PVY strains (PVY^o^, PVY^N^, PVY^NTN^ and PVY^N-Wi^). ELISA testing confirmed that upper, non-inoculated leaves of clone 84.2.P75 became infected with all four PVY strains, whereas clone DB375(1) remained uninfected (Supplementary Table 2). Then, plantlets of 256 individuals from the 08H1 population were manually inoculated with PVY^O^ and the establishment and progression of any virus infection was monitored. None of the inoculated leaves in either susceptible or resistant plants or any of the upper leaves in systemically infected susceptible plants displayed any visible necrosis as evidence of a hypersensitive resistance (HR), although systemic mottling symptoms were apparent in susceptible plants (Supplementary Fig. 1). At 21 days post-inoculation, the upper leaves of the plants were collected and tested by ELISA (tests on all clones were repeated at least twice). We observed 120 plants to be uninfected and so to be resistant and 136 to be infected and thus susceptible to PVY^O^, a segregation ratio that is consistent with an approximate 1:1 ratio (^2^ = 1.0, *P* > 0.1) expected from the segregation of a single dominant resistance gene in the heterozygous configuration in the resistant parent (Supplementary Table 3). These results confirmed our previous findings (Torrance et al. 2009) and further demonstrated that the resistance operated against all of the PVY strains tested.

Plants from the 06H1 population were inoculated with PVY^O^ in the same way as for the 08H1 population. In common with the results obtained with the 08H1 population, none of the inoculated or systemically infected leaves displayed any visible necrosis. ELISA testing at 21 dpi of the upper, non-inoculated leaves showed that 127 plants were PVY-resistant (no virus detected by ELISA) and 24 were PVY-susceptible (virus detected by ELISA with A_405_ values more than twice that of healthy plants; Fenlon and Sopp 1991), a ratio which does not conform to commonly obtained segregation ratios for a single dominant gene in a diploid population (Supplementary Table 4). Therefore, we suspect that there is more than just a single PVY-resistance factor segregating in the 06H1 population. Interestingly, the resistant parent of the 06H1 population (HB171(13)) maintained its PVY resistance at elevated (28 °C) temperature.

### Genetic analysis of 08H1 and 06H1 populations identifies a major source of PVY resistance on chromosome 9

In the absence of any prior genotypic data for the 08H1 population, an initial screen was carried out using previously mapped SSR markers to establish the chromosomal location of the PVY resistance gene. SSRs were selected to give coverage of all 12 potato chromosomes and a bulked segregant analysis (BSA) approach (Michelmore et al. 1991) was used to identify markers linked to resistance or susceptibility. Resistant and susceptible bulks (each consisting of 30 plants) were screened alongside the resistant (DB375(1)) and susceptible (84.2P.75) parents. Results indicated that two SSR markers on chromosome 9 (STM1021 and STM3012) showed a degree of linkage to PVY resistance (data not shown).

Diversity Array Technology (DArT) analysis (Diversity Arrays Technology, Canberra, Australia) carried out on the 08H1 resistant and susceptible parents and the progeny bulks revealed 98 DArT markers to be present in the resistant parent and bulk, and absent from the susceptible parents and bulk. Of the 98 markers for which DNA sequences were available, 66 were unambiguously identified in the DM genomic pseudomolecules (Sharma et al. 2013) and of these, 23 were very clearly anchored to chromosome 9, with the remaining 43 being distributed between the other 11 chromosomes. A BLAST search using the chromosome 9 DArT marker sequences (http:potatogenomics.plantbiology.msu.edu/index.html) anchored them to chromosome 9 (~ 44–52 Mb) distributed across 6 superscaffolds (PGSC0003DMB000000115, PGSC0003DMB000000263, PGSC0003DMB000000407, PGSC0003DMB000000309, PGSC0003DMB000000207, PGSC0003DMB000000298). SSR markers were selected from the sequences of the 6 superscaffolds for further linkage analysis, one of which (SSR651299) was applied to the 08H1 population (178 individuals) and was able to distinguish resistant and susceptible plants. Resistant plants generated PCR products 272 bp and 310 bp in size, whereas susceptible plants generated the 310 bp fragment but not the 272 bp product (data not shown), suggesting that these plants are homozygous for the 310 bp fragment and that the smaller fragment is linked in coupling to the resistance gene present in DB375(1). Of the 85 resistant progeny plants genotyped, 81 showed the presence of the 272 bp fragment, whereas 81 of 93 susceptible plants failed to amplify this fragment, thus confirming linkage between the PVY resistance and this marker allele in the 08H1 population.

**Fig. 1:**
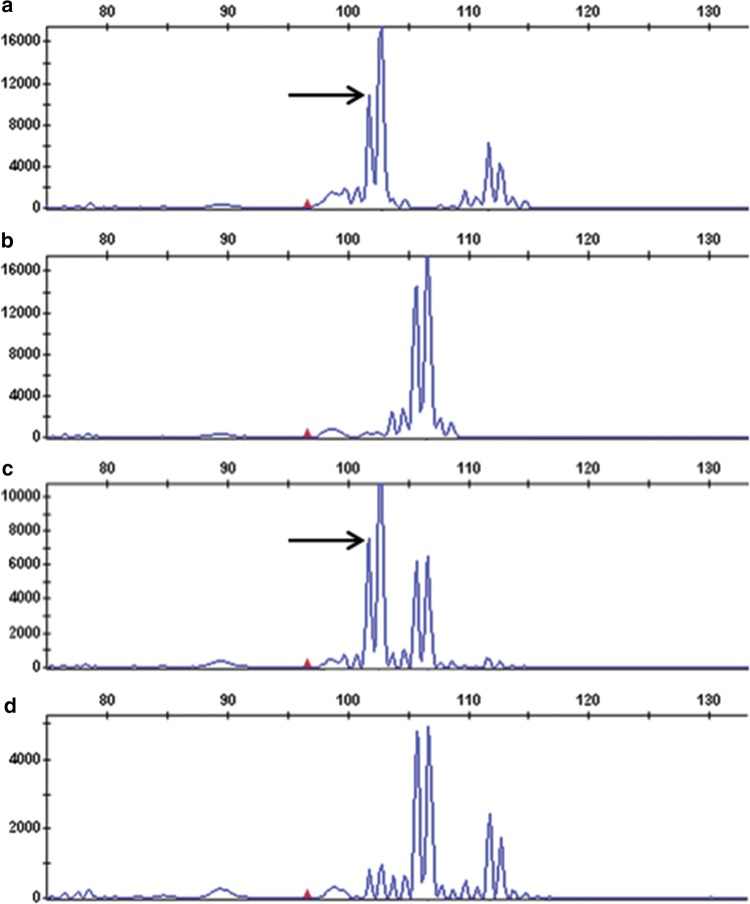
Fluorescent single sequence repeat (SSR) plot showing a 102 bp fragment (arrowed) amplified by primers for PM0360 present in resistant parent (**a**) and resistant bulk (**c**) but absent from susceptible parent (**b**) and susceptible bulk (**d**)

**Fig. 2:**
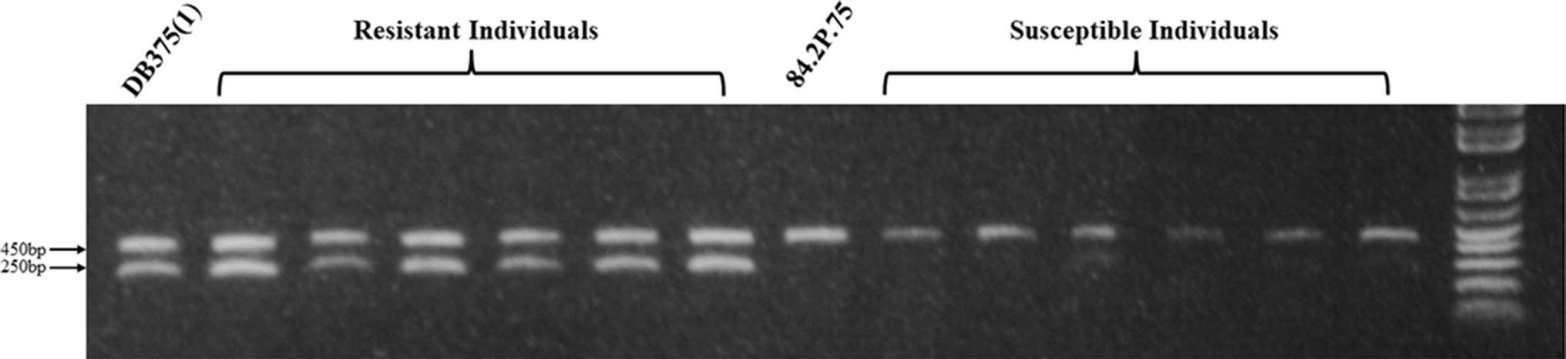
DNA from resistant parent (DB375(1)), resistant individuals, susceptible parent (84.2P.75) and susceptible individuals amplified using T1582 allele-specific primers which amplify a 250 bp fragment (indicated by lower left arrow) only from resistant samples. A 450 bp fragment (upper left arrow) generated from SSR STM5148 was present in all samples and used as an internal PCR control

**Fig. 3:**
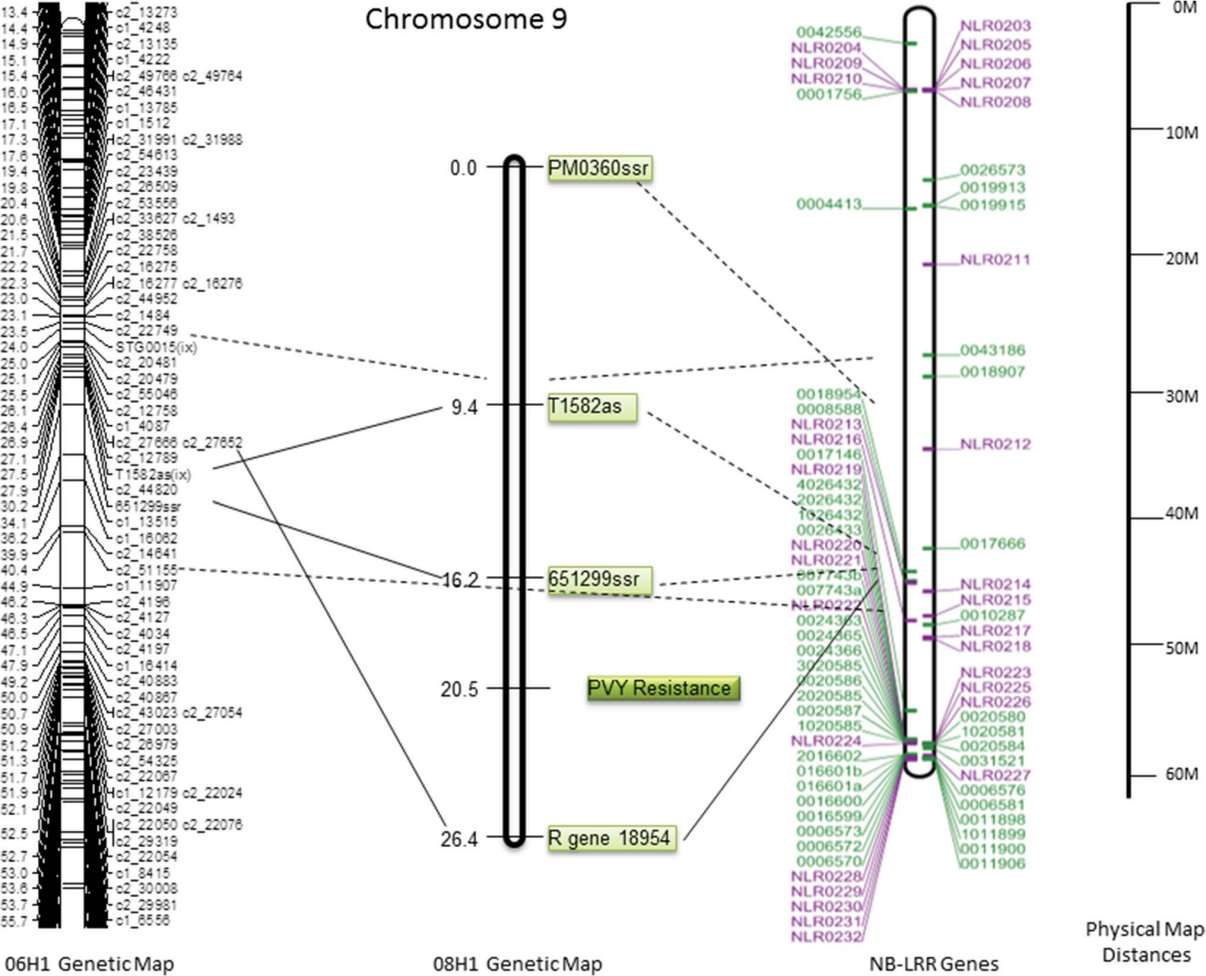
Comparative maps of chromosome 9 produced using genetic data from 06H1 and 08H1 populations and RenSeq analysis. Positions of markers are shown relative to NB-LRR genes ( reproduced from Jupe et al. 2013) and diagnostic markers for PVY resistance phenotype

Additional markers mapping to the same approximate physical position in the DM genome (Sharma et al. 2013) and in the tomato genome (Sato et al. 2012) were tested for polymorphism and two, PM0360 and T1582, were found to be polymorphic in 08H1. PM0360, an SSR marker, has been mapped in the ‘DM × DD’ population (Sharma et al. 2013) on chromosome 9 at 40.486 cM. In the 08H1 cross, the PM0360 primers amplified a 102 bp fragment in the resistant parent and bulk which was absent from the susceptible parent and bulk samples (Fig. 1). T1582 is a COS (Conserved Ortholog Set) marker which mapped to 51 cM on chromosome 9 in the tomato genetic map Tomato-EXPEN 2000 (Fulton et al. 2002). PCR product sequences from the parents of 08H1 showed three SNPs at nucleotide positions 253, 260 and 266 bp which were heterozygous in resistant genotypes and homozygous for one allele in susceptible genotypes (data not shown). Primers (T1582as; Supplementary Table 1) were designed to amplify a 250 bp allele-specific PCR fragment specific to the allele found only in the resistant parent (Fig. 2). SSR STM5148 was used as an internal positive PCR control and amplified a 450 bp fragment. The two markers, T1582 and PM0360, were mapped in relation to the resistance phenotype and were both found to be slightly ‘north’ of SSR marker SSR651299 with respect to the resistance locus, further confirming linkage of the resistance to chromosome 9 in the 08H1 population (Fig. 3).

Examination of the Potato Genome browser (https://potato.plantbiology.msu.edu/cgi-bin/gbrowse/potato/) identified an NB-LRR gene near SSR651299 on Chromosome 9 in genome scaffold PGSC0003DMB400000115. Cloning of this R gene, PGSC0003DMG400018594, from the 08H1 resistant parent DB375(1) revealed that it has a copia sequence insertion within the 5′ region of the gene which would probably render it non-functional. A T/C SNP (homozygous C/C in the susceptible parent (84.2P.75) and T/C in the resistant parent (DB375(1)) were identified in this gene. Primers were designed (Supplementary Table 1) to amplify a 240 bp fragment containing this SNP from 10 susceptible and 10 resistant progeny individuals from the 08H1 population. All 10 resistant progeny were heterozygous T/C for the SNP and 9 out of 10 susceptible progeny were homozygous C/C. Further work was carried out to genotype all individuals within the 08H1 population, and the data generated were used to map the NB-LRR gene PGSC0003DMG400018594 on chromosome 9, ~ 10 cM south of SSR651299 (Fig. 3).

The 08H1 population, being derived from a cross between two closely related Phureja clones, displays a very low level of marker polymorphism, rendering targeted marker development problematic. The highly polymorphic ‘hybrid’ 06H1 population was utilised to further characterise the location of PVY resistance in our Phureja material. Moreover, the 06H1 population has an existing single nucleotide polymorphism (SNP) marker-based linkage map (Prashar et al. 2014). We assumed that due to the close familial relationships among our ‘core’ Phureja clones (they were descended from a narrow set of progenitor group Phureja germplasm accessions), the same resistance factors were likely to be segregating in the two crosses. Three of the four chromosome 9 markers (SSR651299, T1582as and STG0015) which were found to be linked to PVY resistance in 08H1 were added to the 06H1 SNP linkage map (Prashar et al. 2014) (Supplementary Table 5). The nonparametric Kruskal–Wallis (KW) test in MapQTL® 6.0 (Van Ooijen 2009) was performed using values derived from ELISA values (after subtraction of the mean control healthy leaf value) and produced a highly significant KW value of 42.91 for the SSR marker SSR651299 (mapped to ~ 33.3 cM), with a maximum KW value of 48.46 for SNP c2_22749 located 6.8 cM ‘north’ of SSR651299 on linkage group 9 at 26.5 cM.

**Fig. 4:**
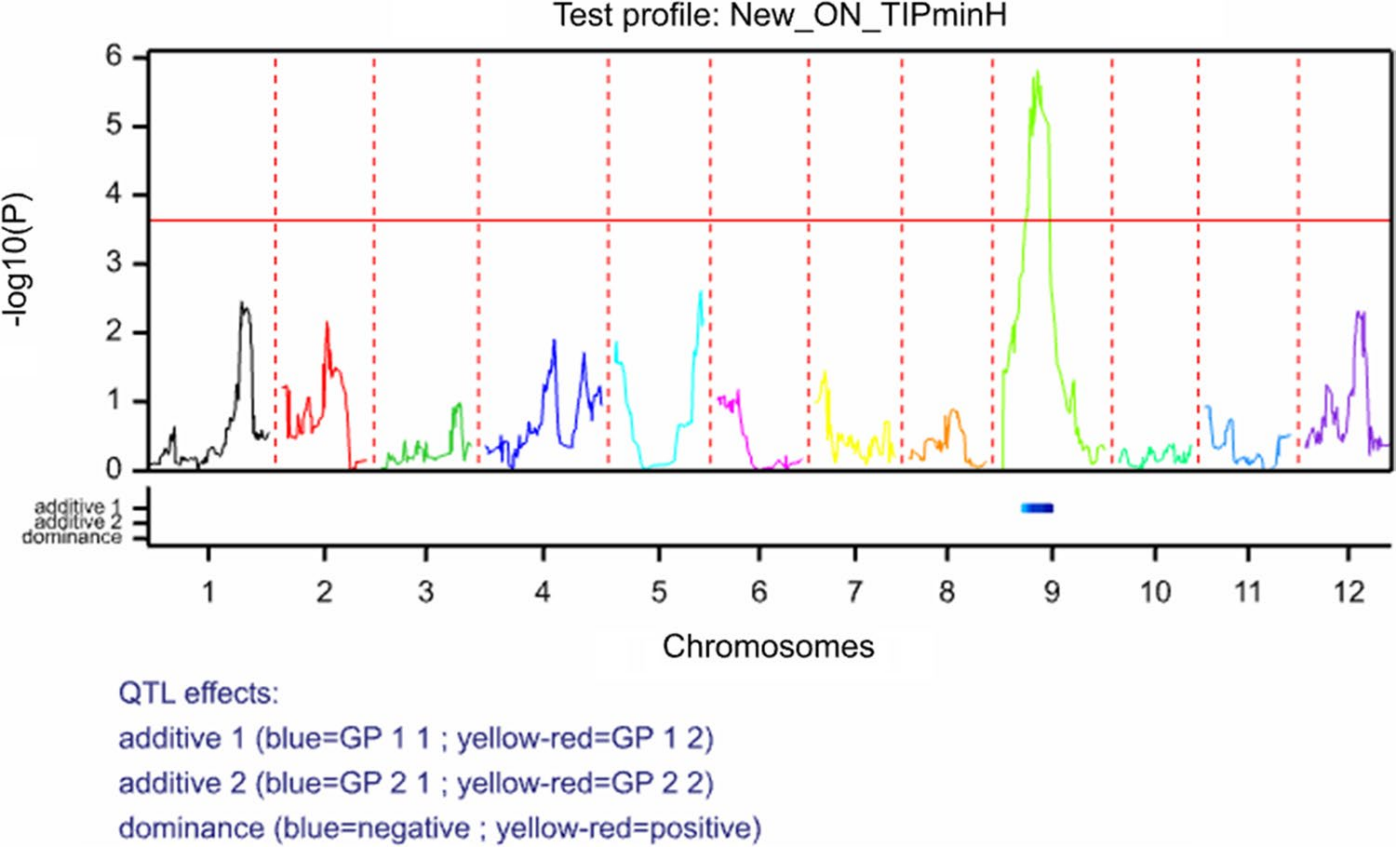
QTL profile plot from Genstat v19 for PVY inoculated leaf ELISA score. The plot shows the -log_10_(P) values across the 12 potato chromosomes. The plot also shows that the main effect detected on chromosome 9 is an additive effect arising in the maternal parent

A composite interval mapping (CIM) approach was adopted using Genstat 19. A ‘single environment single trait’ approach was used, whereby traits were analysed separately for candidate QTLs. The routine DQSQTLSCAN was used, and a genome wide threshold of 0.01 was used to select QTL (–log_10_P value of 4.176 at 1%). Initially, a simple interval mapping (SIM) approach was used with a maximum step size of 20 cM and this identified a large effect QTL on chromosome 9 at the same location as the KW analysis (Fig. 4) with no suggestion of any additional QTL effects on ELISA values, even after using the chromosome 9 marker as a co-factor. The most tightly linked maternal marker is SNP c2_22749 (−log10(*P*) = 6) (which maps to 26.5 cM on chromosome 9, showing perfect correspondence with the nonparametric KW test. A virtually identical result is obtained if the ELISA data are converted to a ‘0/1’ variate (0 = susceptible, 1 = resistant), although the significance level is far higher (−log10(P) = 12.2) using this scoring method and the QTL peak shifts slightly to the location of marker c2_53556 at 24.5 cM. In both QTL analyses, the major QTL effect detected is a large additive effect on the ELISA score (− 0.23 ± 0.05) or resistance score (+ 0.25 ± 0.03), with the main effect on resistance arising from the maternal resistant (HB171(13)) parent as expected.

### RenSeq analysis confirms the chromosome 9 resistance locus and suggests a second putative resistance-associated locus on chromosome 4

The 06H1 population was further analysed by RenSeq to corroborate the mapping results and to locate the PVY resistance locus in relation to the annotated NB-LRR genes in the potato genome (Jupe et al. 2013). RenSeq reads were generated for resistant parent HB171(13), susceptible parent 99.FT.1b5, and resistant and susceptible bulks formed by pooling DNAs from 20 resistant (BR) and 20 susceptible (BS) clones. For the mapping, reads were trimmed and mapped at a 3% mismatch rate against the DM genome (v4.03) (Supplementary Table 6). Reads from the resistant and susceptible parents as well as BR and BS pools were individually merged and filtered for SNPs. SNPs that displayed 0–10% alternative alleles in the susceptible parent and BS and 40–60% alternative alleles in the resistant parent and BR were selected as being significant. A minimum sequence depth of 20-fold was used for the SNP calling. Only SNPs that were independently corroborated in the bulks and parents are reported and locate within two NB-LRRs on Chromosome 4 and four NB-LRRs on Chromosome 9 (Table 1). Single SNPs were identified in RDC0001NLR0049 and RDC0001NLR0055 on Chromosome 4. On Chromosome 9, a single SNP was identified in NB-LRR PGSC0003DMG400018954, two in PGSC0003DMG400008588, five in RDC0001NLR0213 and two in RDC0001NLR0214 (Table 2). The positions of the significant SNPs are depicted in Supplementary Fig. 2. The locus RDC0001NLR0214 is not predicted to encode a full-length NB-LRR. The length and percentage amino acid identity for the remaining five NB-LRRs are presented in Supplementary Table 7.

**Table 1:** SNP analysis for the resistant parent HB171(13), the susceptible parent 99.FT.1b5 and Bulks BR and BS. The number of SNPs conforming to the expected ratio is shown. The number of *R* genes (and their ID) with SNPs that was independently confirmed in the parents and bulks are shown in relation to the 755 NB-LRRs defined in Jupe et al. (2013)

Chromosome	Number of SNPs			Number of NB-LRR genes with SNPs
	Bulks	Parents	Bulks and Parents	
1	0	226	0	0
2	0	344	0	0
3	0	33	0	0
4	3	779	2	2
5	3	412	0	0
6	3	383	0	0
7	0	165	0	0
8	0	247	0	0
9	24	380	10	4
10	0	149	0	0
11	1	400	0	0
12	7	247	0	0
0	0	97	0	0
Total	41	3862	12	6

**Table 2:** SNP analysis for the resistant parent HB171(13), the susceptible parent 99.FT.1b5 and Bulks BR and BS. The position of the SNPs conforming to the expected ratio is shown for *R* genes on Chromosome (chr) 4 and 9. The position of *R* genes corresponds to the 755 NB-LRRs defined in Jupe et al. (2013)

Gene	chr	Start–stop	SNP_name (notation: chr_position)
RDC0001NLR0049	4	7,855,418–7,859,148	ST4.03ch04_7857838
RDC0001NLR0055	4	21,923,841–21,924,629	ST4.03ch04_21923860
PGSC0003DMG400018954	9	45,529,847–45,536,832	ST4.03ch09_45530995
PGSC0003DMG400008588	9	46,312,335–46,316,463	ST4.03ch09_46313335 ST4.03ch09_46315075
RDC0001NLR0213	9	46,458,710–46,462,684	ST4.03ch09_46458943 ST4.03ch09_46459013 ST4.03ch09_46460183 ST4.03ch09_46461358 ST4.03ch09_46461761
RDC0001NLR0214	9	47,147,352–47,150,352	ST4.03ch09_47147541 ST4.03ch09_47147798

The genomic positions of the SNPs detected using Renseq were compared with the locations of the most significant chromosome 9 SNP markers detected by genetic analysis. Thus, the most significant SNP marker (c2_22749) maps to ~ 31.6 Mb on chromosome 9, whereas the Renseq-derived SNPs map to 45.5–46.3 Mb. Overall these different analyses correlate quite well, given that the Renseq capture probes are not randomly distributed in the potato genome due to the uneven distribution of NB-LRR genes in the DM potato genome sequence. This is especially true for chromosome 9 where there are very few NB-LRR genes in the DM genome at the locus harbouring the major PVY resistance gene detected in the two populations.

The identification of SNPs using Renseq linked to resistance on chromosome 4 is suggestive of the presence of a second locus in 06H1 plants that was not detected using standard QTL approaches. Inspection of graphical genotype data for chromosome 4 using the 06H1 linkage map marker data did reveal a very slight but statistically insignificant marker genotype distortion for the SNP marker c2_48868 (~ 13 cM) in the 39 resistant plants that lack the relevant chromosome 9 resistance genotype (26 of 39 plants are heterozygous for c2_48868; however, the 24 fully susceptible plants show a completely balanced segregation pattern of 12 heterozygous: 12 homozygous). Clearly this observation and the Renseq data require further investigation as the presence of a second locus on chromosome 4 is, at this moment, not conclusively demonstrated.

### Resistant plants support virus infection in a small number of epidermal cells

**Fig. 5:**
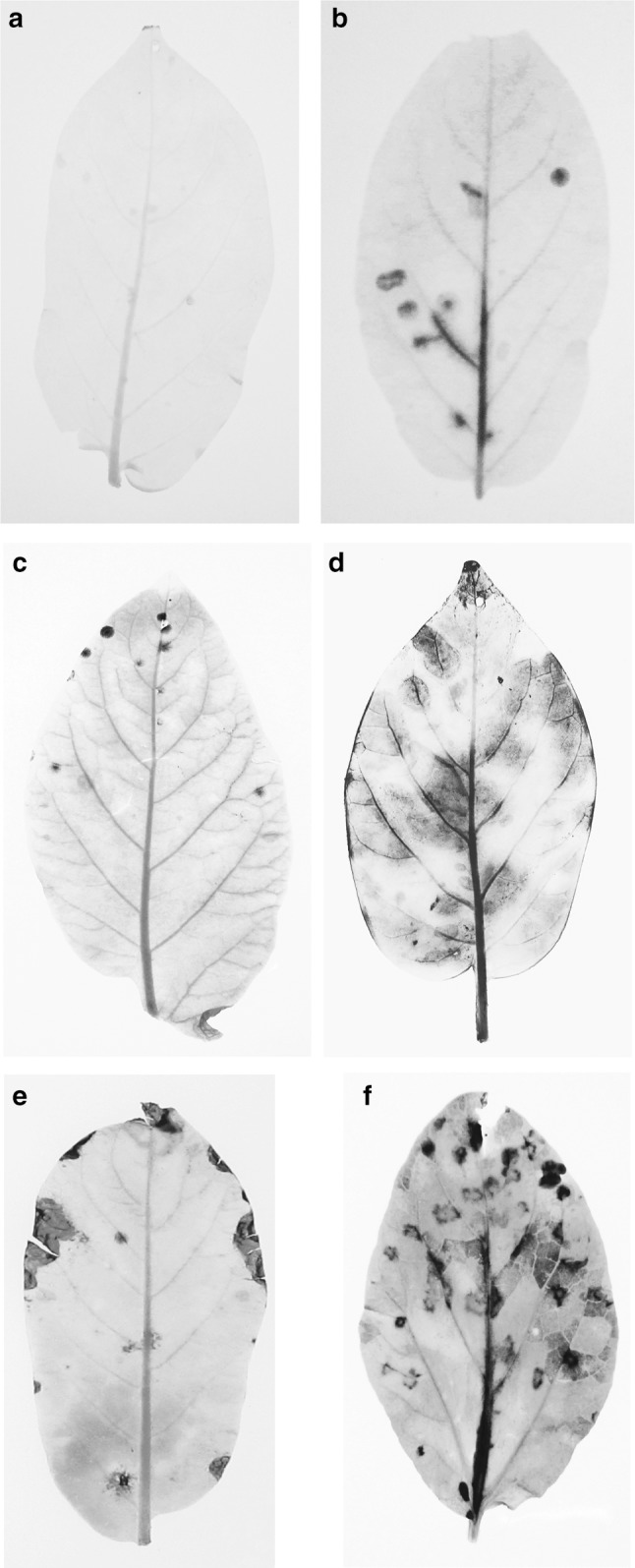
Potato leaves showing blue staining produced after manual inoculation with PVY-GUS. Blue patches of β-glucuronidase stain, indicating virus replication, are visible. **a** cv Tacna; **b** cv Corine; and clones **c** DB375(1); **d** 84.2P.75; **e** HB171(13) and **f** 99.FT.1b5

To gain insight into the mechanism of resistance, plants were mechanically inoculated with PVY engineered to express the GUS protein and both virus-inoculated and upper, non-inoculated leaves were assayed for GUS production. Multiplication and movement of the virus within the plant is revealed by the production of a dark blue staining in the areas of the leaf where virus infection has occurred. Virus infection of the parents of the 08H1 and 06H1 crosses was compared with that in PVY-resistant potatoes cv Tacna (carrying Ry_adg_; Ortega and Lopez-Vizcon 2012) and cv Corine (carrying Ry_sto_; Valkonen et al. 2017). No necrotic local lesions typical of a hypersensitive response were observed in any of the inoculated leaves of susceptible or resistant plants (Supplementary Fig. 1). Representative images of GUS staining of inoculated leaves are presented in Fig. 5. GUS staining (and thus PVY infection) was clearly visible as irregular and expanding patches that spread into leaf veins and the midrib of the susceptible parents, 99.FT.1b5 and 84.2P.75, showing the establishment of locally expanding areas of infection and indicating the initiation of the process of systemic spread of the virus. Systemic infection of the upper (tip) leaves in both susceptible parental lines by PVY-GUS was subsequently demonstrated, confirming that the PVY-GUS clone behaved similarly to wild type (non-recombinant) PVY (Supplementary Fig. 3).

For the resistant parents of both the 08H1 and 06H1 crosses, DB375(1) and HB171(13), a few, small blue patches (mean diameter of 1.45 mm) became visible in the inoculated leaves (mean number per leaf, 3 and 0.7, respectively) but no GUS staining of leaf veins was observed and no systemic movement occurred (Fig. 5). Leaves of cv Corine (Ry_sto_) showed a similar pattern, with a few blue patches developing (mean number 7 per leaf) and with occasional spread of the virus into the leaf veins, although the virus did not progress into upper leaves. For cv Tacna (Ry_adg_), no GUS staining was apparent anywhere in the inoculated or upper, uninoculated leaves, suggesting that this resistance prevents even small amounts of PVY replication and cell-to-cell spread.

## Discussion

### A new source of PVY resistance

In this paper, we report the identification and genetic characterisation of a previously unreported major gene on potato chromosome 9 acting to confer resistance to PVY in three biparental crosses, 05H1, 08H1 and 06H1. In the latter cross, the availability of a dense SNP-based linkage map has allowed a more detailed genetic analysis. A gene on chromosome 9 confers resistance to systemic infection by PVY and restricts infection in the initial inoculated leaf to small groups of cells from which the infection fails to develop. We suggest this gene be named henceforth as Ry(o)_*phu*_. The novelty of this locus has been confirmed by checking markers shown to be tightly linked to PVY resistance in previous publications. We can detect no linkage with such markers and can also confirm that the gene reported here maps considerably less distally (i.e. 40–50 Mb) than those reported in other recent studies (Sato et al. 2006; Tomczynska et al. 2014), which appear to be located nearer to the distal tip of chromosome 9, around 59–61 Mb. The RenSeq data are suggestive of a second genetic effect on chromosome 4 associated with PVY resistance in the 06H1 cross, although, the presence of this locus could not be verified by conventional QTL analysis using ELISA or qualitative ‘0/1’ resistance scores.

### Predicted R genes linked to the chromosomal locations of the mapped resistance factor on chromosome 9

Many examples of host resistance to viruses and other plant pathogens have been shown to be due to the action of NB-LRR resistance genes, for example R*x* and Ry_sto_, both genes from potato, provide resistance to Potato virus X and PVY, respectively (Bendahmane et al. 1999; Grech-Baran et al. 2019). Although the determination of more genome sequences from different potato species is in progress, the most complete published sequence is for the doubled monoploid clone DM1-3 516 R44 of *S. tuberosum* group Phureja (referred to as DM). DM is susceptible to PVY (unpublished data) and so lacks the functional resistance gene(s) that we are investigating in our study. Nevertheless, the map location of the chromosome 9 PVY resistance gene in the 06H1 population suggests that it resides in a different genetic location to other published pathogen resistances on this linkage group, such as Ry_chc_ and Rpi-moc1 (Sato et al. 2006; Simko et al. 2007). Initially, 438 NB-LRR-type resistance (R) genes were identified in DM potato, which was later increased to 755 by use of a RenSeq capture sequencing approach (Xu et al. 2011; Jupe et al. 2013). The latter analysis predicted the existence of 75 NB-LRR genes on chromosome 9, of which 10 are clustered near the proximal end of the chromosome (nucleotides 2,075,540–6,015,882) and 56 are clustered in the distal region of the chromosome (nucleotides 45,529,847–61,028,633). This clustering of ~ 88% of the NB-LRR genes at the extremities of chromosome 9 representing only about 35% of the total chromosome length is typical of overall resistance gene distribution in many plant species and which has been noted in potato (Sharma et al. 2013). The genetic mapping described here identified the SNP marker c2_22749 as being most closely linked to the chromosome 9 PVY resistance phenotype. This marker is located at nucleotide 31,630,568 in chromosome 9, within the central region of the chromosome that is very sparsely populated with predicted NB-LRR genes.

RenSeq analysis identified a different set of NB-LRR derived SNPs on chromosome 9 that were associated with PVY resistance. Thus, a single SNP was identified in NB-LRR PGSC0003DMG400018954, two in PGSC0003DMG400008588, five in RDC0001NLR0213 (Jupe et al. 2013) and two in RDC0001NLR0214 (Jupe et al. 2013). These loci (45.5–47.1 Mb) are located significantly “south” of the c2_22749 marker (~ 31.6 Mb) showing the tightest genetic linkage to the PVY resistance allele. This apparent discrepancy appears to be due to the highly non-random distribution of target loci in the RenSeq probe capture library as well as the relatively high physical to genetic distance ratios in this region of chromosome 9, whereby markers show only slightly lower KW values but are physically located much closer to the RenSeq markers. For example, SNP marker c2_44820 shows KW values of ~ 42 and physically maps to 46.7 Mb which is located in the exact genomic region containing the markers detected by RenSeq on chromosome 9.

Chromosome 4 of DM contains a total of 126 NB-LRR genes (Jupe et al. 2013), and clusters are distributed along the chromosome. Although Renseq analysis identified two NB-LRRs as potentially being associated with PVY resistance in the 06H1 plants, we have been unable to corroborate this using QTL studies.

### Non-NBS-LRR-type genes known to be involved in virus resistance

In addition to the NB-LRR genes, there are some examples in other studies where other types of gene have been found to provide dominant resistance to plant viruses (reviewed in de Ronde et al. 2014; Calil and Fontes 2017). These include the RTM1, RTM2 and RTM3 Arabidopsis genes that prevent systemic movement of viruses that are similar to PVY (Chisholm et al. 2000; Whitham et al. 2000), Ty-1 a tomato RNA dependent RNA polymerase that inhibits Tomato yellow leaf curl virus (Verlaan et al. 2013) and Tm-1 a tomato protein that acts against Tomato mosaic virus by physically binding to the virus replication proteins and preventing their functioning (Ishibashi et al. 2007).

As well as the putative NB-LRR genes mentioned above, the region on chromosome 9 containing the resistance gene contains several other genes that could possibly be involved in virus infection and might contribute to PVY resistance. These include SGS3 (a suppressor of gene silencing protein that is known to interact with potyvirus VPg [Rajamaki et al. 2014]), a translation initiation factor and several heat shock family proteins (some of which are known to be necessary for virus replication [Hafren et al. 2010; Freire 2014]). However, our analysis considers an NB-LRR gene(s) to be the most likely resistance factor.

### The resistance does not completely prevent virus replication

R gene-mediated virus resistance in potato has been classified into two types (Valkonen 2015). One is hypersensitive resistance (HR) where visible areas of necrosis develop in inoculated leaves. This type of resistance is often virus strain specific and may also be less effective at raised temperatures, sometimes leading to necrosis and collapse of systemic infected leaves (Lukan et al. 2018). A second form of resistance is known as extreme resistance (ER) where very little or no visible necrosis is seen following virus inoculation. This resistance usually protects against all strains of the virus. However, as discussed by van Eck et al. (2017), currently it is not known whether the different resistance phenotypes reflects differences in the direct function of the ER-type (e.g. Ry) and HR-type (e.g. Ny) NB-LRR genes or is caused by allelic variation in the downstream genetic pathways that these NB-LRR gene connect to.

Several studies have been done to try to understand the mechanism of PVY ER provided by the *Ry*_*sto*_ gene. For example, individual protoplast cells of potato cultivars carrying the *Ry*_*sto*_ gene and showing extreme resistance at the level of the whole leaf, nevertheless allowed virus replication and expression of the PVY CP to the same degree as did protoplasts from susceptible cultivars (Hinrichs et al. 1995). This work was followed by a study using similar plants and either PVY or the related virus tobacco etch virus (TEV) that expressed the GUS protein (Hinrichs et al. 1998). Here, they found that these viruses replicated in both the initially infected cell and a few (6–10) adjacent cells but no systemic infection was ever detected. Small necrotic streaks (2–4 mm) were seen on the underside of PVY inoculated leaves in the collenchyma cells, which are structural support cells lying under the epidermis and often adjacent to the vascular cambium, but no necrosis was seen in the vascular system itself. With TEV-GUS small blue areas were seen in extreme resistant cultivars, whereas GUS expression extended into the major veins of the inoculated leaf of the susceptible cultivar. Using the cloned *Ry*_*sto*_ gene, transgenic potato plants showed no visible symptoms after inoculation with PVY and no virus was detected in upper leaves by RT-PCR (Grech-Baran et al. 2019). However, a strong HR response was seen after transient expression of Ry_sto_ in PVY-infected *N. benthamiana* plants and in *Ry*_*sto*_-transgenic *N. tabacum* plants that were inoculated with PVY, suggesting that the plant genetic background and perhaps also the expression level of the *Ry*_*sto*_ gene might affect the strength and nature of the defence reaction. One further observation from this study was that the visible HR reaction to PVY in a potato cultivar carrying the *Ny*-1 resistance gene is abolished by incorporation of the *Ry*_*sto*_ gene as a transgene, i.e. that *Ry*_*sto*_ is epistatic to *Ny*-1.

In our tests, no visible hypersensitive response or necrotic symptoms were apparent on the inoculated leaves or other parts of the Phureja plants, suggesting the resistance is of the ER type. A PVY clone expressing GUS produced visible blue patches of infection on susceptible clones that spread to the veins and, thereafter, to upper leaves but the resistant clones displayed only one or very few small foci in inoculated leaves and failed to develop any spreading infection. In comparison, no blue patches were ever observed on cv Tacna that carries *Ry*_*adg*_, and in cv Corine that carries *Ry*_*sto*_ there were more blue patches (approx. 7 per leaf) than were seen in our resistant phureja clones and also a limited movement of virus into leaf veins. From these observations, it would seem that the Phureja resistance mechanism is a little stronger acting than the Corine resistance but weaker to operate than the Tacna resistance. Interestingly, the Ry_sto_ resistance in Corine originated from use of the CPC 2093 accession by breeders in The Netherlands, and this clone has also provided PVY resistance to the cultivars Santé and Festien (Valkonen et al. 2017). Both Santé and Corine are considered to deliver extreme resistance, with no visible necrotic/hypersensitive reaction, whereas in Festien the resistance was proposed to be of the HR type, with pinpoint lesions forming in systemic leaves developing into necrosis of the small veins when PVY was delivered to test plants by grafting (van Eck et al. 2017). This reinforces the idea that the physical manifestation of the resistance derived from any particular NB-LRR gene is strongly influenced by other genes controlling signalling and defence response pathways downstream of the initial pathogen recognition step that is overseen by the NB-LRR gene. Previously, Corine was challenged by mechanical inoculation of PVY, by grafting and by infection of mesophyll cell protoplasts using purified PVY RNA (Barker and Harrison 1984). In these experiments, no necrotic symptoms developed following mechanical or graft inoculation, and PVY replication was detected in only a very low percentage of protoplasts, suggesting that the ER mechanism was functional in the majority of the isolated cells. Our finding that the resistance in Corine did not prevent a limited replication and cell-to-cell spread of the virus PVY-GUS, albeit with the absence of visible necrosis, might reflect the very high sensitivity of the GUS staining assay compared with the virus detection methods used in the earlier study. These findings show that, similar to what was reported for *Ry*_*sto*_, the resistance in the 06H1 and 08H1 Phureja plants did not prevent virus replication in and around the initially inoculated epidermal cells but prevented entry to the vascular system and, thereafter, long distance virus movement.

In conclusion, we have discovered broad spectrum PVY resistance ultimately derived from Group Phureja clones in the Commonwealth Potato Collection (https://www.hutton.ac.uk/about/facilities/commonwealth-potato-collection). The clones included in the initial screen (Torrance et al. 2009), some of which represent parental material used in this study, were from the Scottish Crop Research Institute’s ‘long day adapted’ Phureja population which was derived via mass selection for tuberisation under long days (Carroll 1982). Virus resistance in Phureja potatoes is particularly valuable because, for example, in Kenya (and other potato growing regions of sub-saharan Africa) their low dormancy is a highly desired property since, like Andean farmers, they grow two (or three) crops per year corresponding to the rainy seasons. The variety Mayan Gold, which also contains the PVY resistance described in this paper, was introduced to Kenya and passed National Performance trials in 2014. In discussions, with local stakeholders, this variety was valued because of its very acceptable taste and because it cooked quickly, thereby, providing demonstrable savings in energy costs for consumers. Moreover, we have found that farmer self-saved seed can remain PVY-free over six field generations, the maximum duration tested (H. Were and L. Torrance, unpublished), greatly reducing costs for farmers of buying commercially-produced virus-tested seed potatoes.

The 08H1 cross used in our mapping studies is a ‘pure Group Phureja’ cross, whereas 06H1 is a Phureja-Tuberosum hybrid cross, and this latter cross has been better characterised so is perhaps a better source for introgressing the newly discovered resistance factor(s) into breeding material. Clones from the 06H1 population have several other well-characterised attributes, apart from PVY resistance, that make them potentially useful parents in breeding programmes. Traits that segregate in the population include late blight resistance and tuber dormancy (Glenn Bryan, unpublished data), heat tolerance (Trapero-Mozos et al. 2018) and tuber shape (Prashar et al. 2014).

## Electronic supplementary material

Below is the link to the electronic supplementary material.

Supplementary Figure 1. Manual inoculation of potato leaves with PVY does not produce any visible necrosis but can produce systemic mottling. Panels a-d, inoculated leaf; panel e, systemic leaf; (a) HB171(13), (b) cv. Tacna, (c) phureja 84.2.P75, (d) cv. Corine, (e) 06H1 clone (93) (TIF 6854 kb)

Supplementary Figure 2. Graphical representation of RenSeq sequences that contain informative SNPs linked to the novel PVY resistance. Chromosomes 1-12 are depicted on the x-axis and the numbers of informative SNPs within a 1Mb interval are shown as dots on chromosome 4 (two SNPs in two individual genes) and chromosome 9 (10 SNPs in four individual genes). Shaded in the background are the numbers of genes that were assessed at each locus and represent the position of NB-LRRs used for the bait library design (EPS 1943 kb)

Supplementary Figure 3. PVY-GUS can infect susceptible potato plants systemically. Panel (a) phureja 84.2.P75, (b) 99FT.1b5 (EPS 13947 kb)

Supplementary Table 1. Primers sequences (DOCX 14 kb)

Supplementary Table 2. Reaction of resistant and susceptible parents of the O8H1 and O6H1 crosses to four isolates of PVY (DOC 35 kb)

Supplementary Table 3. ELISA data for PVY inoculation of 08H1 population (XLSX 15 kb)

Supplementary Table 4. ELISA data for PVY inoculation of 06H1 population (XLSX 16 kb)

Supplementary Table 5. Graphical genotyping data for mapping Resistant versus Susceptible phenotype in the 06H1 population (XLSX 838 kb)

Supplementary Table 6. RenSeq read data (DOCX 14 kb)

Supplementary Table 7. The predicted length and percentage amino acid identity of the five full-length NB-LRRs identified by RenSeq (DOCX 14 kb)
