# Supplementary figures and images for: Natural resistance to Potato virus Y in Solanum tuberosum Group Phureja
Source: Theor Appl Genet. 2020 Jan 16;133(3):967–80. doi: 10.1007/s00122-019-03521-y (PMC7021755; doi:10.1007/s00122-019-03521-y)

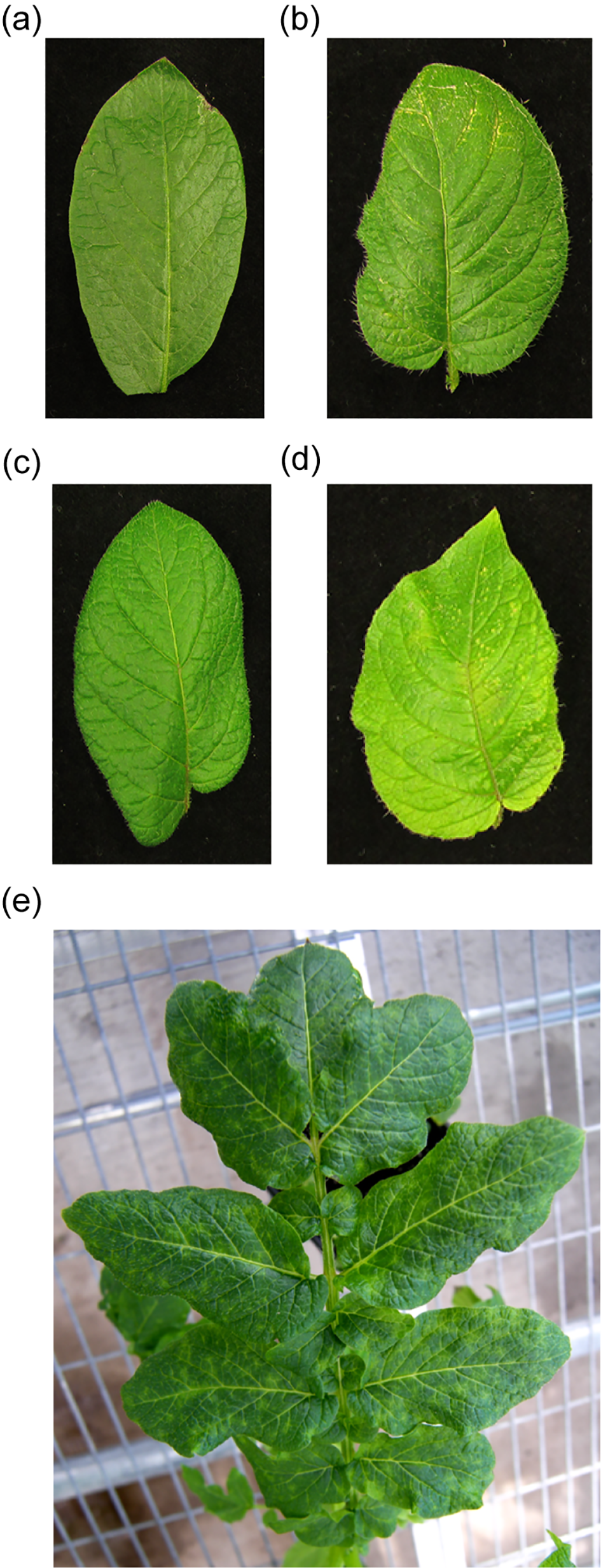

Supplement: Supplementary file 1 — Supplementary Figure 1. Manual inoculation of potato leaves with PVY does not produce any visible necrosis but can produce systemic mottling. Panels a-d, inoculated leaf; panel e, systemic leaf; (a) HB171(13), (b) cv. Tacna, (c) phureja 84.2.P75, (d) cv. Corine, (e) 06H1 clone (93) (TIF 6854 kb) [file 122_2019_3521_MOESM1_ESM.tif]
