# Supplementary material for: Natural resistance to Potato virus Y in Solanum tuberosum Group Phureja
Source: Theor Appl Genet. 2020 Jan 16;133(3):967–80. doi: 10.1007/s00122-019-03521-y (PMC7021755; doi:10.1007/s00122-019-03521-y)
Supplement: Supplementary file 4 — Supplementary Table 1. Primers sequences (DOCX 14 kb) [file 122_2019_3521_MOESM4_ESM.docx]

Table S1. Primer sequences used for PCR of genomic DNA

| Marker | Forward Primer | Reverse Primer |
| --- | --- | --- |
| 651299ssr | TTGTCATTATTTTATTTCTTGCTTGC | TCCGTTTTTATTTTAATTGGCATT |
| PM0360 | TCCCCCAAATTTTACTTTGAAACAAG | TTATTTTCATGCCACAAAGTAGCG |
| T1582as | TAACAGCTGTCAGAAACTCG | GAAAGCTTGTAAAAATACG |
| RT-PCR | GGACAAACAAAAGGGGAAAA | AACCAAACGATCCTCAATCG |
