# Supplementary material for: Natural resistance to Potato virus Y in Solanum tuberosum Group Phureja
Source: Theor Appl Genet. 2020 Jan 16;133(3):967–80. doi: 10.1007/s00122-019-03521-y (PMC7021755; doi:10.1007/s00122-019-03521-y)
Supplement: Supplementary file 5 — Supplementary Table 2. Reaction of resistant and susceptible parents of the O8H1 and O6H1 crosses to four isolates of PVY (DOC 35 kb) [file 122_2019_3521_MOESM5_ESM.doc]

Supplementary Table 2. Reaction of resistant and susceptible parents of the O8H1 and O6H1 crosses to four isolates of PVY

|  | **O8H1 parents**  **842.P75 DB375(1)** | |  | **O6H1 parents**  **99.FT.1b5 HB171(13)** |
| --- | --- | --- | --- | --- |
| **PVY^O^** | + | - |  | + - |
| **PVY^NTN 390^** | + | - |  | + - |
| **PVY^N-Wi^** | + | - |  | n/d n/d |
| **PVY^N^** | + | - |  | + - |

+: virus detected in upper, non-inoculated leaves by ELISA

-: virus not detected in upper, non-inoculated leaves by ELISA
