# Supplementary material for: Natural resistance to Potato virus Y in Solanum tuberosum Group Phureja
Source: Theor Appl Genet. 2020 Jan 16;133(3):967–80. doi: 10.1007/s00122-019-03521-y (PMC7021755; doi:10.1007/s00122-019-03521-y)
Supplement: Supplementary file 9 — Supplementary Table 6. RenSeq read data (DOCX 14 kb) [file 122_2019_3521_MOESM9_ESM.docx]

**Table S6.** The total number of reads following RenSeq are shown for the resistant parent HB171(13), the susceptible parent 99.FT.1b5 and Bulks BR and BS. The reads were mapped allowing for a 3% mismatch rate against the 755 NB-LRRs defined in Jupe et al., 2013.

| Sample | Reads | Mismatch rate | Reads mapped against DM v.4.03  NB-LRRs (Jupe et al 2013) | | |
| --- | --- | --- | --- | --- | --- |
|  |  |  | Total | % | Coverage of RenSeq DM |
| HB171(13) | 2248448 | 3 | 1056656 | 46.99 | 41.18 |
| 99.FT.1b5 | 2459722 | 3 | 1118458 | 45.47 | 41.51 |
| Resistant (BR) | 1318804 | 3 | 612450 | 46.44 | 23.69 |
| Susceptible (BS) | 2493908 | 3 | 1164680 | 46.7 | 42.73 |
