# Supplementary material for: Natural resistance to Potato virus Y in Solanum tuberosum Group Phureja
Source: Theor Appl Genet. 2020 Jan 16;133(3):967–80. doi: 10.1007/s00122-019-03521-y (PMC7021755; doi:10.1007/s00122-019-03521-y)
Supplement: Supplementary file 10 — Supplementary Table 7. The predicted length and percentage amino acid identity of the five full-length NB-LRRs identified by RenSeq (DOCX 14 kb) [file 122_2019_3521_MOESM10_ESM.docx]

|  | Length (aa) | A | B | C | D | E |
| --- | --- | --- | --- | --- | --- | --- |
| A-RDC0001NLR0049 | 887 |  | 24 | 26 | 21 | 26 |
| B-RDC0001NLR0055 | 841 | 24 |  | 23 | 24 | 23 |
| C-PGSC0003DMG400018954 | 993 | 26 | 23 |  | 20 | 31 |
| D-PGSC0003DMG400008588 | 1032 | 21 | 24 | 20 |  | 20 |
| E-RDC0001NLR0213 | 1272 | 26 | 23 | 31 | 20 |  |

Supplementary Table 7. The predicted length and percentage amino acid identity of the five full-length NB-LRRs identified by RenSeq.
